# A yoga program for cognitive enhancement

**DOI:** 10.1371/journal.pone.0182366

**Published:** 2017-08-04

**Authors:** Devon Brunner, Amitai Abramovitch, Joseph Etherton

**Affiliations:** Department of Psychology, Texas State University, San Marcos, Texas, United States of America; University Children's Hospital Tuebingen, GERMANY

## Abstract

**Background:**

Recent studies suggest that yoga practice may improve cognitive functioning. Although preliminary data indicate that yoga improves working memory (WM), high-resolution information about the type of WM subconstructs, namely maintenance and manipulation, is not available. Furthermore, the association between cognitive enhancement and improved mindfulness as a result of yoga practice requires empirical examination. The aim of the present study is to assess the impact of a brief yoga program on WM maintenance, WM manipulation and attentive mindfulness.

**Methods:**

Measures of WM (Digit Span Forward, Backward, and Sequencing, and Letter-Number Sequencing) were administered prior to and following 6 sessions of yoga (*N* = 43). Additionally, the Mindfulness Attention Awareness Scale was administered to examine the potential impact of yoga practice on mindfulness, as well as the relationships among changes in WM and mindfulness.

**Results:**

Analyses revealed significant improvement from pre- to post- training assessment on both maintenance WM (Digit Span Forward) and manipulation WM (Digit Span Backward and Letter-Number Sequencing). No change was found on Digit Span Sequencing. Improvement was also found on mindfulness scores. However, no correlation was observed between mindfulness and WM measures.

**Conclusions:**

A 6-session yoga program was associated with improvement on manipulation and maintenance WM measures as well as enhanced mindfulness scores. Additional research is needed to understand the extent of yoga-related cognitive enhancement and mechanisms by which yoga may enhance cognition, ideally by utilizing randomized controlled trials and more comprehensive neuropsychological batteries.

## Introduction

There has been significant interest in cognitive enhancement in recent years, including several investigations of the potential cognitive benefits of computerized cognitive training (e.g., [1, 2]). Several variables may promote this trend, including the increased availability of cognitive programs; extended life span and subsequent increase in the prevalence of elderly experiencing cognitive decline; and the increased prevalence of attention deficit/hyperactivity disorder (ADHD) [3, 4]. However, a recent meta-analysis on the efficacy of computer-based cognitive training programs indicates minimal effects on cognition and behavior [5, 6].

For these reasons, researchers have begun to explore cognitive enhancement via Mind-Body Practices (e.g., mindfulness training [MT], yoga, meditation). Preliminary studies suggest that yoga practice may result in improved cognitive performance, among other potential benefits in healthy adults [7–10]. Indeed, a meta-analysis of both short- and long- term effects indicates that yoga practice is associated with improvement in cognitive functioning generally in both long-term (Hedges’ *g* = 0.33) and short-term studies (Hedges’ *g* = 0.56), with medium effect sizes reported in short-term studies’ measures of attention and processing speed (Hedges’ *g* = 0.49) and executive functioning (Hedges’ *g* = 0.39). However, as the limited number of studies and the heterogeneous use of cognitive tests indicate, evidence for cognitive improvement via yoga practice should be considered preliminary [11]. As such, additional research on the possible efficacy of yoga for cognitive improvement is needed.

## Working memory

WM is a limited-capacity cognitive system that involves the temporary storage, processing and manipulation of information [12] and is involved in a number of cognitive functions [13, 14], including reasoning, decision-making, learning, and behavior [15, 16]. WM is considered a part of the central executive function domain and may subserve all executive functions. Indeed, the WM system plays a central role in a diverse array of cognitive tasks, and WM capacity shares significant variance with measures of fluid intelligence [17, 18]. A useful conceptualization of WM subdomains differentiates between maintenance WM, involving short-term storage of information; and manipulation WM, involving both short-term storage and manipulation of task-relevant information. The Wechsler Adult Intelligence Scale (WAIS) Digit Span Forward task is a gold standard measure to assess maintenance WM. The WAIS Digit Span Backwards, and more so the WAIS Letter- Number Sequencing tasks [13], are frequently used to assess manipulation WM. The recent edition of the WAIS introduced the Digit Span Sequencing–a new test added to the Digit Span subtest. This subtest is associated with manipulation WM functions, but has been introduced only relatively recently, and limited published peer-reviewed psychometric work has yielded inconsistent results [19, 20].

### Yoga, mindfulness, & cognition

Yoga is a derivative of Indian ancient science, but the physical practice and way of life has become very popular in Western countries [21–23]. The physical yoga discipline includes asanas (postures), pranayama (breathing techniques), and dhyana (meditation) [24]. Tools to withdraw the senses (pratyahara), concentrate the mind (dharana), and develop unwavering awareness (dhyana) manifest from dedicated yoga practice [25]. Yoga practice comprises not just stretching, but rather dynamic movements tied to the breath. Indeed, yoga is associated with multiple health benefits including increased physical stamina, balance, flexibility, and relaxation [26]. However, yoga also appears to offer potential psychological benefits through the inclusion of mindfulness training, involving the practice of meditation as well as the dynamic combination of proprioceptive and interoceptive awareness [27]. The regular practice of mindfulness skills results in both awareness and profound focus by drawing attention to the present moment without judgment.

The physical and cognitive benefits associated with yoga and mindfulness [28] may be due to mechanisms including pranayama and activation of the parasympathetic nervous system [29]; meditative or contemplative practices [30, 31]; increased body perception [32]; stronger functional connectivity within the basal ganglia [33]; or increased activation of grey matter volume and amygdala with regional enlargement [34, 35]. The meditative aspect of yoga practice may contribute to the enhancement or improvement of WM, although the mechanism is yet to be clarified. Subramanya and Telles [8] utilized the Wechsler Memory Scale (WMS): Digit Span Forward and Backward to assess WM (*N* = 57) in healthy adults pre- and post- yoga-based relaxation techniques (cyclic meditation and supine rest). They found significantly improved WMS scores following both yoga-based relaxation techniques. Additionally, a randomized trial with healthy adults (*N* = 42) found that a single session of a yogic meditation technique significantly enhanced WM task scores [36]. Repeated practice of attentional focus and redirecting attention may be a possible active ingredient for developing greater attentional control [37, 38]. However, to our knowledge, no study to date has examined the potential relationship between mindfulness and WM cognitive function in the context of yoga practice, and no available literature exists on maintenance versus manipulation WM in this context.

The current study examined whether 6 sessions of yoga training would result in improvement in WM performance as well as improvement in self-reported mindfulness. We hypothesized that yoga training would result in improved WM performance on outcome measures assessing both maintenance WM and manipulation WM. It was further hypothesized that improvements in mindfulness would be associated with improvements in WM performance.

## Materials & methods

### Measures

#### WAIS IV: Digit span

The Wechsler Adult Intelligence Scale, 4th edition (WAIS-IV) [39] subtest Digit Span (DS) is a widely-used measure of WM [40, 41]. The DS test includes: Digit Span Forward (DSF), in which the participant is read a sequence of numbers and recalls the numbers back in the same order, assessing maintenance WM; Digit Span Backwards (DSB), in which the participant recalls the numbers in the reverse order, assessing a higher-load manipulation WM; and Digit Span Sequencing (DSS), a new subtest introduced in the WAIS-IV that is presumed to assess maintenance WM, in which the participant recalls the numbers in ascending order. However, while the first two subtests have been extensively investigated and have demonstrated very good psychometric properties, the latter subtest has received relatively little attention, and recent reports indicate some weaknesses in terms of Digit Span Sequencing test’s psychometric properties [19, 20]. Nevertheless, we chose to administer the third subtest in order to be able to extract an overall DS Total score (DST), which incorporates scores from all three subtests.

#### WAIS IV: Letter-number sequencing

The WAIS-IV subtest Letter-Number Sequencing (LNS) is a well-validated measure of manipulation WM [42]. The participant is read a series of numbers and letters and asked to recall the numbers in ascending order, followed by the letters in alphabetical order.

#### The Mindful Attention Awareness Scale (MAAS)

The MAAS is a 15-item scale designed to assess dispositional mindfulness, which refers to a receptive awareness or attention to what is taking place in the present moment [43]. Items are scores on a 6-point Likert scale ranging from 1 = almost always to 6 = almost never. All items are first person statements such as “It seems I am ‘running on automatic,’ without much awareness of what I’m doing.”, and “I could be experiencing some emotion and not be conscious of it until sometime later.” Thus, higher scores reflect elevated mindful awareness. The MAAS demonstrated strong psychometric properties, with good (α = 0.84) to very good (α = 0.91) internal consistency found across different age groups and countries [44, 45]. A number of studies have shown that the MAAS assesses a unique quality of consciousness that is associated with enhanced self-awareness as well as a variety of wellbeing constructs [46, 47]. The MAAS’s Cronbach alpha for the present study indicated good internal consistency (first administration α = 0.84; second administration α = 0.81).

### Participants

Forty-three participants (8 males, 35 females) were recruited by contacting instructors of psychology and exercise science classes in addition to recruitment announcements in the fitness center at Texas State University. Inclusion criteria included being 18 years old or older as well as affirming a statement regarding general good physical health and absence of physical conditions that would interfere with participation in the yoga program. No other inclusion/exclusion criteria were imposed. Participant mean age was 24.77 (*SD* = 10.72), with an age range between 18 and 80. Notably, 95% of our participants were between 18–36 years old, with one participant aged 57 and another aged 80 years old. Most participants (91%) were undergraduate students, and four participants had an undergraduate degree. Of the entire sample, 51% (*n* = 22) reported no past yoga experience. This study was approved by the Texas State University Institutional Review Board, and all participants provided signed informed consent. In addition to free participation in the present yoga program, participants received as incentive a free one-semester exercise pass (valued at $60) for campus recreation classes.

### Procedures

A within-subjects design was used to assess changes in cognitive functioning and mindfulness following yoga training. Participants completed baseline assessment of maintenance and manipulation WM (DS and LNS) as well as mindfulness (MAAS) up to 1 week before participating in yoga sessions. They were then allowed to select one of two formats of the 6-session yoga program: one 60-minute yoga session per week for 6 weeks, or two 60-minute yoga sessions per week for 3 weeks (only two participants chose the latter). Participants met with the experimenter (DB) individually in a research assessment room to complete a short demographic questionnaire, complete the MAAS questionnaire, and be administered the DS and the LNS at time 1, prior to attending yoga sessions, and time 2, at least 1 day after completing yoga sessions. Given scheduling constraints, assessment at time 2 took place within two weeks of the final yoga session (*MDays* = 6.32, *SD* = 3.61, range = 13). The study was conducted between September and December 2015, and all yoga sessions were held on campus at the Department of Campus Recreation facilities.

#### Yoga intervention

Hatha yoga employed in this study included asanas connected with pranayama and a 10-minute guided mindfulness meditation in supine rest. The mindfulness meditation exercise included elements of focused attention meditation, which encouraged participants to direct and sustain attention to the breath and focus on the inhalation and exhalation sensations, cueing to detect mind wandering or distractors (e.g., “allow thoughts to pass by like floating clouds in the sky”), and to bring the attention back to the breath. Influenced by Iyengar yoga, guidance emphasized precision of proper body alignment in the performance of the asanas and pranayama as well as redirecting focus on the breath. Mats, blocks, and straps were provided to all participants. All sessions lasted 60 minutes and were led by the first author, who has over a decade of experience in yoga practice, and who has completed 500-hour certification as a therapeutic yoga teacher. Classes were offered 4 times/week for participants to fulfill their selected program requirements.

During each session, traditional yogic philosophy elements of non-violence (ahisma) and truthfulness (satya), apart from the five other yamas, or yogic ethical guidelines, were emphasized. When moving through the asanas, honesty and non-violence cueing was advocated for safe practice. These two yamas were highlighted during each session as a reminder of being aware of the body’s physical limitations both on and off the mat. Additionally, the asanas were held for approximately five breaths. The class structure included a warm-up breathing exercise with directed observation and scanning of mental and physical state in the present moment as well as choosing to set a positive affirmation or something to be thankful for in the present moment (Table 1). Followed by asana practice, every yoga session ended with a guided mindfulness meditation in supine rest. Home practice was not required, but participants were asked to document and provide information about home practice prior to every subsequent yoga class.

**Table 1:** Sample 60-minute yoga session construction.

Component	Approximate Duration
**Seated mindfulness scan and set positive affirmation**	3–5 min
**Seated asanas**	6–9 min
1. Spinal twists 2. Staff pose 3. Head-to-knee forward bend 4. Reclining hand-to-big-toe pose	
**Standing asanas**	25–27 min
5. Mountain pose 6. Tree pose 7. Warrior II pose 8. Extended side angle stretch 9. Half-moon pose 10. Standing forward bend 11. Warrior I pose 12. Downward facing dog pose	
**Restorative asanas**	6–8 min
13. Cobra pose 14. Child’s pose 15. Cat-cow pose 16. Eye-of-the-needle pose 17. Legs up the wall	
**Final relaxation: guided mindfulness meditation**	10 in
18. Corpse pose (supine rest)	

Each asana was held for approximately five breaths.

## Results

### Preliminary moderator analyses

In order to assess the potential impact of age, gender, previous yoga experience, and hours practicing yoga outside of the study, we computed the scaled score difference (time 2—time 1) for the five outcome measures (DSF, DSB, DSS, DST, and LNS), which were then examined via multivariate analysis of variance and Pearson correlation analyses with potential moderators. No overall difference in score change was found between males and females across WM outcome measures (Wilk’s Lambda = 0.910, *F*(5, 37) = 0.735, *p* = 0.602), and no gender difference was found for any single measure (all *p’s* 0.12–0.82). During the study, 25 participants reported practicing yoga outside the study program. No overall difference in score change was found between participants who practiced yoga outside the study sessions and those who did not (*F*(5, 37) = 0.509, *p* = 0.768), and no effect was found for any single measure (all *p’s* 0.37–0.96). To assess dose response among the 25 participants who did practice yoga outside study sessions, we conducted Pearson’s correlations between the number of hours practiced outside the study sessions and change on the five outcome measures. No significant correlation was found between these variables (*r*’s 0.04–0.25, *p*’s 0.11–0.97).

An overall difference in score change was found between individuals with or without previous yoga experience (*F*(5, 37) = 3.673, *p* = 0.008, Partial *η*^2^ = 0.33), where a larger improvement was found in individuals with past yoga experience. Subsequent univariate analyses revealed that this significant difference stems from a significant difference found on the LNS test (*F*(1,41) = 10.683, *p* = 0.002, Partial *η*^2^ = 0.21), where individuals with past yoga experience exhibiting significantly greater improvement. No other univariate differences were found. However, in order to take a more conservative approach, previous yoga experience was controlled for in all subsequent analyses. Notably, only 3 participants chose the 2 yoga sessions × 3 weeks yoga program, and only 4 participants completed an undergraduate degree (all other participants were undergraduate students). These sample sizes prevented testing of the moderating effect of these variables.

#### Main analyses

Repeated measures ANOVAs were conducted to assess the difference on the five outcome measures while incorporating previous yoga experience as a between group factor. A significant improvement was found on the DSF score (*F*(1, 41) = 6.375, *p* = 0.016, Partial *η*^2^ = 0.13). No significant interaction effect was found for treatment × past yoga experience (*p* = 0.43). A significant improvement was found on the DSB score (*F*(1, 41) = 13.251, *p* = 0.001, Partial *η*^2^ = 0.24). No significant interaction effect was found for treatment × past yoga experience (*p* = 0.067). No significant improvement was found on the DSS score (*F*(1, 41) = 1.570, *p* = 0.217, Partial *η*^2^ = 0.037), and no significant interaction effect was found for treatment × past yoga experience (*p* = 0.267). However, a significant improvement was found on the DST score (*F*(1, 41) = 21.217, *p* < 0.0001, Partial *η*^2^ = 0.35), and no significant interaction effect was found for treatment × past yoga experience (*p* = 0.768).

**Fig 1:**
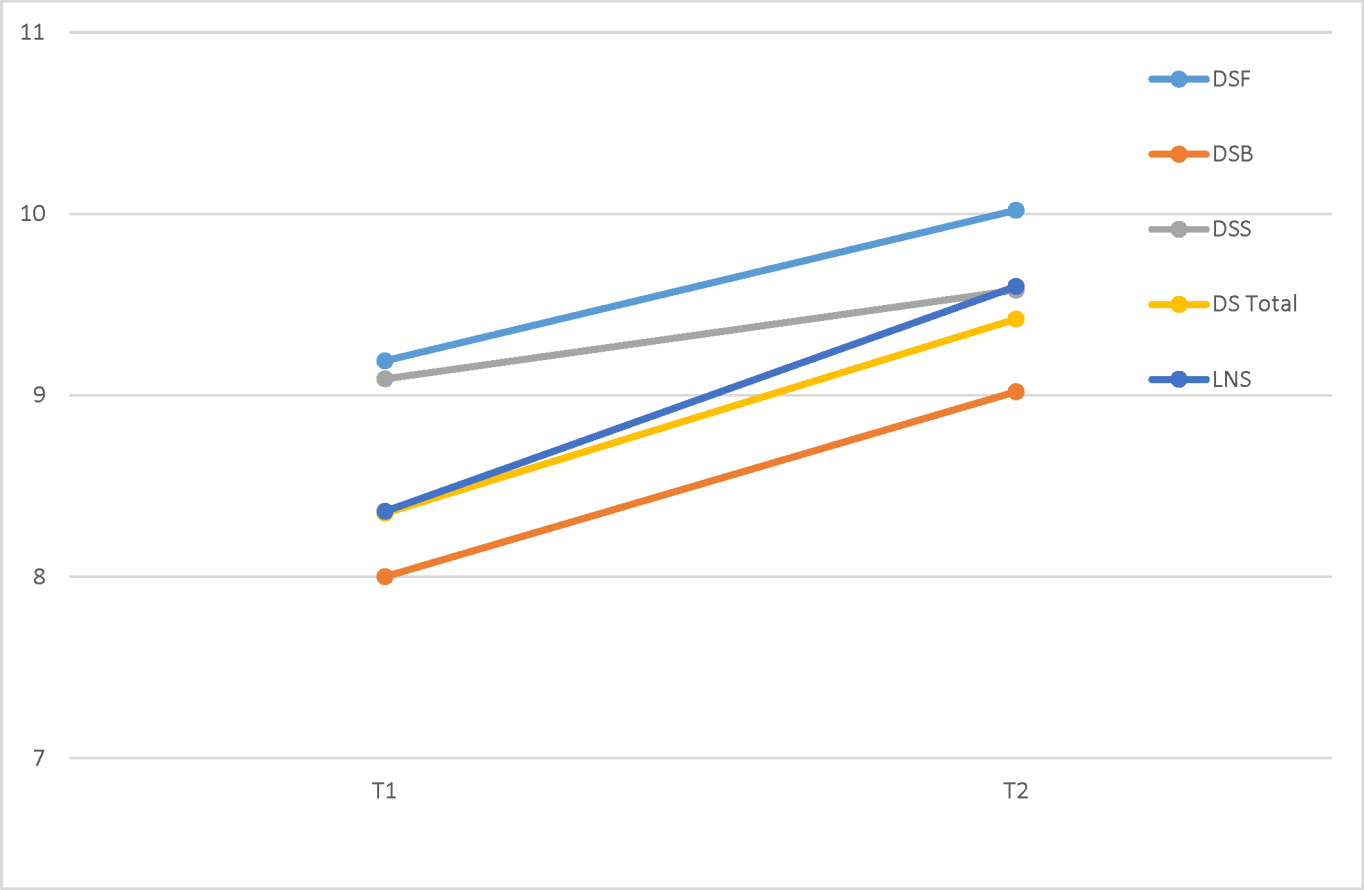
Test scores on working memory indices before and after yoga intervention. DSF = Digit Span Forward; DSB = Digit Span Backward; DSS = Digit Span Sequencing; DS Total = Digit Span Total; LNS = Letter-Number Sequencing.

A significant improvement was found on the LNS score (*F*(1, 41) = 23.683, *p* < 0.0001, Partial *η*^2^ = 0.37), and a significant interaction effect was found for treatment effect × past yoga experience (*F*(1, 41) = 12.152, *p* = 0.0001, Partial *η*^2^ = .23). Subsequently, individual repeated measures conducted separately among participants with and without past yoga experience showed that a significant improvement on the LNS was found only for the group with previous yoga experience (*F*(1, 19) = 25.126, *p* < 0.0001, Partial *η*^2^ = 0.57), and not for those with no previous yoga experience (*F*(1, 21) = 1.418, *p* = 0.247, Partial *η*^2^ = 0.06). Given that it is plausible that a practice effect exists as part of the improvements on these tests, we present the means and standard deviations from a study examining practice effects on the WAIS-IV in 3- and 6- month retest intervals with no intervention between test sessions [48]. As presented in Table 2, partial *η*^2^ effect sizes indicate that participants in the current study demonstrated greater improvement in both DS and LNS performance from pre- to post- training testing than was observed in Estevis and colleagues’ test-retest study [48]. See Fig 1 for a graphic illustration of these results. Notably, Pearson’s correlations between the scaled score improvement on the four tasks and baseline performance yielded three negative significant correlations for DFS (*r* = -0.43, *p* = 0.004), DSS (*r* = -0.49, *p* = 0.001), and LNS (*r* = -0.45, *p* = 0.003), where lower scaled scores were associated with a greater increase following yoga training. Subsequent Fisher’s Z transformation and comparison between participants with and without previous yoga experience yielded no significant differences between the two groups pertaining to these associations.

**Table 2:** Working memory function before and after yoga intervention.

	Time 1		Time 2				
	*M*	*SD*	*M*	*SD*	*M*_*diff*_	*F*(1,42)	Partial *η*^2^
*DS Forward*	9.19	3.18	10.02	20.98	0.83	6.37**	0.13
*DS Backward*	8.00	1.50	9.02	2.18	1.02	13.25***	0.24
*DS Sequencing*	9.09	2.50	9.58	2.53	0.49	1.57	0.04
*DS Total*	8.35	2.09	9.42	2.30	1.07	21.71***	0.35
*LNS*	8.36	2.05	9.60	2.08	1.54	23.68***	0.37
*MAAS*	3.56	0.76	4.09	0.62	0.53	23.43***	0.36
***DS***^***a***^	10.3	2.8	10.8	3.2	0.5	6.13*	0.10
***LNS***^***a***^	10.9	3.2	11.2	2.7	0.3	0.85	0.02

*M*, Mean; *SD*, standard deviation; *M*_*diff*,_ Mean difference; *ES*, Effect size (partial *η*^2^); *DS*, Digit Span; *LNS*, Letter-Number Sequencing; *MAAS*, Mindfulness Attention Awareness Scale.

^**a**^ *DS* and *LNS* test-retest comparison sample scores (*N* = 54) from Estevis et al. [48].

(Level of significance:

*** *p* < .001

** *p* < .01

* *p* < .05)

In order to assess change in mindfulness, we conducted a repeated measure ANOVA between the two administrations of the MAAS. Results revealed a significant change (in the direction of increased mindfulness) on this scale (*F*(1, 42) = 23.432, *p* < 0.0001, Partial *η*^2^ = 0.36). Moderator analyses including age, gender, past yoga experience, and additional yoga practice revealed no significant moderating effect (all *p’s* > 0.05). Furthermore, change in the MAAS score was not correlated with change in any of the cognitive outcomes (all *r*’s < 0.074, all *p’*s > 0.64).

## Discussion

In the current study, we examined whether six 60-minute sessions of yoga training, which included mindfulness meditation practice, improved WM functions and mindfulness. In accordance with our primary hypothesis, completion of this program was associated with overall significant improvement in measures of WM. Specifically, participants significantly improved performance on a maintenance WM task (DSF) over and above previous yoga experience. Participants also improved on two manipulation WM tasks (i.e., DSB, and LNS). Notably, improvement on the LNS was found only among participants with previous yoga experience. Given that among the tasks administered LNS is considered a task with the highest cognitive load, it may be the case that participants with previous yoga experience were able to benefit more from the yoga program. This may be supported by our results indicating that additional yoga practice during the program did not moderate any WM effects. Thus, it appears that both previous guided practice and present guided practice had an effect on WM, where the former was important in the most demanding task.

Effect sizes for WM improvement were found to be large, indicating that the improvement in the current study was more substantial than that attributable to practice effects [48]. Surprisingly, participants did not demonstrate improvement on one manipulation WM, namely the DSS subtest. However, compared to the strong psychometric properties of the traditional DS and LNS tasks, this subtest of the WAIS-IV Digit Span test is considered problematic in terms of its psychometric properties [19, 20]. In accordance with our second hypothesis, we found a significant improvement in attentive mindfulness from pre- to post- yoga training as measured by the MAAS questionnaire (corresponding to a large effect size). However, in contrast to our hypothesis, improvement in mindfulness was not associated with any change in cognitive functions. This outcome is similar to another study that found no significant correlations between MAAS and WM task performance pre- to post- mindfulness training [49]. Speculatively, it is possible that yoga enhances WM function without any conscious effort on the part of participants, but mindfulness requires active conscious effort to be present and alert to one’s surroundings [50]. These potentially different mechanisms may explain how yoga would improve both WM and mindfulness independently, such that measures involving these processes may be uncorrelated. Overall, our results are consistent with previous studies that reported improved WM and executive function performance following yoga training [51–56]. Furthermore, incorporating mindfulness practice in the present yoga program results in increased mindfulness that has been associated with increased WM function [37, 38], increased wellbeing, decreased stress, and decreased mood disturbance [30, 40]. Although the specific mechanism underlying the positive effect yoga practice has on WM is unclear, previous research documents that alongside a host of psychological and physiological benefits of yoga [28], yoga practice leads to substantial neurobiological and psychophysiological alterations. Some studies indicate that yoga practice enhances parasympathetic system activity [29], while others report increased gray matter following yoga practice [35]. Most notably, yoga has been found to enhance resting state activation across multiple cortico-striatal neuronal loops [33], a system thought to subserve executive functions [57], and for which alterations have been associated with a host of neuropsychiatric disorders [58].

Limitations of the current study include the absence of a control group and relatively brief study duration. However, comparison of the current results with test-retest data allows for a quasi-control group and demonstrated that greater improvement was observed following yoga practice than was observed in individuals taking DS and LNS twice with no intervening training. In addition, the present sample included a majority of female participants, which may potentially limit generalizability. However, gender was not found to have a significant effect on the present study’s outcome measures. Notably, previous yoga experience was recorded as a binary nominal variable, and not a continuous variable exemplifying the degree of previous experience. However, in light of our results demonstrating the importance of previous yoga experience on the cognitive benefit from yoga, future studies should assess this variable with higher resolution (e.g., length of previous training, type of yoga etc.). Furthermore, we were unable to assess differences in both education and program enrollment type due to limited variability from participants, and we did not screen our participants for neuropsychiatric conditions, although overall baseline performance was in the normative range.

## Conclusions

The results of this study suggest that a brief yoga program incorporating mindfulness mediation may offer an improvement in WM functioning and enhance attentive mindfulness. The current study adds to the limited amount of research available regarding yoga training as a potential method for cognitive enhancement. Future studies should ideally employ a randomized control trial, incorporating broader neuropsychological batteries to enhance our understanding regarding the strength and extent of the cognitive enhancing effects of yoga. Furthermore, although our group’s baseline cognitive performance was in the normative range, future studies should include samples associated with deficient cognitive functioning and psychopathology.

## Supporting information

S1 File Dataset. (SAV)

## References

[1] Geyer J, Insel P, Farzin F, Sternberg D, Hardy JL, Scanlon M, et al Evidence for age-associated cognitive decline from Internet game scores. Alzheimer's & Dementia: Diagnosis, Assessment & Disease Monitoring. 2015;1(2):260–7. doi: 10.1016/j.dadm.2015.04.002 27239508 10.1016/j.dadm.2015.04.002 PMC4876906

[2] Hardy JL, Nelson RA, Thomason ME, Sternberg DA, Katovich K, Farzin F, et al Enhancing Cognitive Abilities with Comprehensive Training: A Large, Online, Randomized, Active-Controlled Trial. PloS one. 2015;10(9):e0134467 doi: 10.1371/journal.pone.0134467 26333022 10.1371/journal.pone.0134467 PMC4557999

[3] Rodgers WL, Ofstedal MB, Herzog AR. Trends in scores on tests of cognitive ability in the elderly U.S. population, 1993–2000. The journals of gerontology Series B, Psychological sciences and social sciences. 2003;58(6):S338–46. Epub 2003/11/14. . 14614121 10.1093/geronb/58.6.s338

[4] Parker MG, Thorslund M. Health trends in the elderly population: getting better and getting worse. The Gerontologist. 2007;47(2):150–8. 17440120 10.1093/geront/47.2.150

[5] Melby-Lervag M, Hulme C. Is working memory training effective? A meta-analytic review. Dev Psychol. 2013;49(2):270–91. Epub 2012/05/23. doi: 10.1037/a0028228 . 22612437 10.1037/a0028228

[6] Melby-Lervag M, Redick TS, Hulme C. Working Memory Training Does Not Improve Performance on Measures of Intelligence or Other Measures of "Far Transfer": Evidence From a Meta-Analytic Review. Perspect Psychol Sci. 2016;11(4):512–34. Epub 2016/07/31. doi: 10.1177/1745691616635612 ; PubMed Central PMCID: PMCPMC4968033. 27474138 10.1177/1745691616635612 PMC4968033

[7] Rocha K, Ribeiro A, Rocha K, Sousa M, Albuquerque F, Ribeiro S, et al Improvement in physiological and psychological parameters after 6months of yoga practice. Consciousness and cognition. 2012;21(2):843–50. doi: 10.1016/j.concog.2012.01.014 22342535 10.1016/j.concog.2012.01.014

[8] Subramanya P, Telles S. Effect of two yoga-based relaxation techniques on memory scores and state anxiety. Biopsychosoc Med. 2009;3:8 Epub 2009/08/14. doi: 10.1186/1751-0759-3-8 ; PubMed Central PMCID: PMCPMC2734564. 19674483 10.1186/1751-0759-3-8 PMC2734564

[9] Nangia D, Malhotra R. Yoga, cognition and mental health. Journal of the Indian Academy of Applied Psychology. 2012;38(2):262–9.

[10] Subramanya P, Telles S. Effect of two yoga-based relaxation techniques on memory scores and state anxiety. BioPsychoSocial Medicine. 2009;3(1):8. 19674483 10.1186/1751-0759-3-8 PMC2734564

[11] Gothe NP, McAuley E. Yoga and Cognition: A Meta-Analysis of Chronic and Acute Effects. Psychosomatic medicine. 2015;77(7):784–97. Epub 2015/07/18. doi: 10.1097/PSY.0000000000000218 . 26186435 10.1097/PSY.0000000000000218

[12] Conway ARA, Cowan N, Bunting MF, Therriault DJ, Minkoff SRB. A latent variable analysis of working memory capacity, short-term memory capacity, processing speed, and general fluid intelligence. Intelligence. 2002;30(2):163–83. http://dx.doi.org/10.1016/S0160-2896(01)00096-4.

[13] Kane MJ, Hambrick DZ, Tuholski SW, Wilhelm O, Payne TW, Engle RW. The Generality of Working Memory Capacity: A Latent-Variable Approach to Verbal and Visuospatial Memory Span and Reasoning. Journal of Experimental Psychology: General. 2004;133(2):189–217. doi: 10.1037/0096-3445.133.2.189 15149250 10.1037/0096-3445.133.2.189

[14] Wiley J, Jarosz AF, Cushen PJ, Colflesh GJ. New rule use drives the relation between working memory capacity and Raven's Advanced Progressive Matrices. Journal of experimental psychology Learning, memory, and cognition. 2011;37(1):256–63. Epub 2011/01/20. doi: 10.1037/a0021613 . 21244117 10.1037/a0021613

[15] Evans JSBT. In two minds: dual-process accounts of reasoning. Trends in Cognitive Sciences. 2003;7(10):454–9. http://dx.doi.org/10.1016/j.tics.2003.08.012. 14550493 10.1016/j.tics.2003.08.012

[16] Anderson JR. Learning and memory. 2000.

[17] Jaeggi SM, Buschkuehl M, Jonides J, Perrig WJ. Improving fluid intelligence with training on working memory. Proceedings of the National Academy of Sciences of the United States of America. 2008;105(19):6829–33. Epub 2008/04/30. doi: 10.1073/pnas.0801268105 ; PubMed Central PMCID: PMCPMC2383929. 18443283 10.1073/pnas.0801268105 PMC2383929

[18] Klingberg T. Training and plasticity of working memory. Trends in Cognitive Sciences. 2010;14(7):317–24. http://dx.doi.org/10.1016/j.tics.2010.05.002. doi: 10.1016/j.tics.2010.05.002 20630350 10.1016/j.tics.2010.05.002

[19] Theiling J, Petermann F. Neuropsychological Profiles on the WAIS-IV of Adults With ADHD. Journal of attention disorders. 2016;20(11):913–24. Epub 2014/01/23. doi: 10.1177/1087054713518241. 24448224 10.1177/1087054713518241

[20] Young JC, Sawyer RJ, Roper BL, Baughman BC. Expansion and re-examination of Digit Span effort indices on the WAIS-IV. The Clinical neuropsychologist. 2012;26(1):147–59. Epub 2012/01/25. doi: 10.1080/13854046.2011.647083 . 22268525 10.1080/13854046.2011.647083

[21] Saper HB, Eisenberg DM, Davis RB, Culpepper L, Phillips RS. Prevalence and patterns of adult yoga use in the United States: results of a national survey. Alternative Therapies in Health & Medicine. 2004;10(2). 15055093

[22] McCall MC. In search of yoga: Research trends in a western medical database. International journal of yoga. 2014;7(1):4 doi: 10.4103/0973-6131.123470 25035601 10.4103/0973-6131.123470 PMC4097914

[23] Khalsa S. Yoga as a therapeutic intervention. Principles Pract Stress Manage. 2007;3:449–62.

[24] Iyengar BK, Menuhin Y. Light on Yoga: The Bible of Modern Yoga: Knopf Doubleday Publishing Group; 1996.

[25] Iyengar BK. Core of the yoga sutras. HarperThorsons, London; 2012.

[26] Tran MD, Holly RG, Lashbrook J, Amsterdam EA. Effects of Hatha Yoga Practice on the Health-Related Aspects of Physical Fitness. Preventive cardiology. 2001;4(4):165–70. Epub 2002/02/08. . 11832673 10.1111/j.1520-037x.2001.00542.x

[27] Mehling WE, Price C, Daubenmier JJ, Acree M, Bartmess E, Stewart A. The Multidimensional Assessment of Interoceptive Awareness (MAIA). PloS one. 2012;7(11):e48230 Epub 2012/11/08. doi: 10.1371/journal.pone.0048230 ; PubMed Central PMCID: PMCPMC3486814. 23133619 10.1371/journal.pone.0048230 PMC3486814

[28] Woodyard C. Exploring the therapeutic effects of yoga and its ability to increase quality of life. International Journal of Yoga. 2011;4(2):49–54. doi: 10.4103/0973-6131.85485 22022122 10.4103/0973-6131.85485 PMC3193654

[29] Jerath R, Edry JW, Barnes VA, Jerath V. Physiology of long pranayamic breathing: neural respiratory elements may provide a mechanism that explains how slow deep breathing shifts the autonomic nervous system. Medical hypotheses. 2006;67(3):566–71. Epub 2006/04/21. doi: 10.1016/j.mehy.2006.02.042 . 16624497 10.1016/j.mehy.2006.02.042

[30] Carmody J, Baer RA. Relationships between mindfulness practice and levels of mindfulness, medical and psychological symptoms and well-being in a mindfulness-based stress reduction program. Journal of behavioral medicine. 2008;31(1):23–33. Epub 2007/09/28. doi: 10.1007/s10865-007-9130-7 . 17899351 10.1007/s10865-007-9130-7

[31] Salmon P, Lush E, Jablonski M, Sephton SE. Yoga and Mindfulness: Clinical Aspects of an Ancient Mind/Body Practice. Cognitive and Behavioral Practice. 2009;16(1):59–72. http://dx.doi.org/10.1016/j.cbpra.2008.07.002.

[32] Impett EA, Daubenmier JJ, Hirschman AL. Minding the body: Yoga, embodiment, and well-being. Sexuality Research & Social Policy. 2006;3(4):39–48. doi: 10.1525/srsp.2006.3.4.39

[33] Gard T, Taquet M, Dixit R, Holzel BK, Dickerson BC, Lazar SW. Greater widespread functional connectivity of the caudate in older adults who practice kripalu yoga and vipassana meditation than in controls. Frontiers in human neuroscience. 2015;9:137 Epub 2015/04/09. doi: 10.3389/fnhum.2015.00137 ; PubMed Central PMCID: PMCPMC4360708. 25852521 10.3389/fnhum.2015.00137 PMC4360708

[34] Desai R, Tailor A, Bhatt T. Effects of yoga on brain waves and structural activation: A review. Complementary therapies in clinical practice. 2015;21(2):112–8. Epub 2015/04/01. doi: 10.1016/j.ctcp.2015.02.002 . 25824030 10.1016/j.ctcp.2015.02.002

[35] Hernandez SE, Suero J, Barros A, Gonzalez-Mora JL, Rubia K. Increased Grey Matter Associated with Long-Term Sahaja Yoga Meditation: A Voxel-Based Morphometry Study. PloS one. 2016;11(3):e0150757 doi: 10.1371/journal.pone.0150757 26938433 10.1371/journal.pone.0150757 PMC4777419

[36] Saoji A, Mohanty S, Vinchurkar SA. Effect of a Single Session of a Yogic Meditation Technique on Cognitive Performance in Medical Students: A Randomized Crossover Trial. Journal of religion and health. 2016 Epub 2016/02/06. doi: 10.1007/s10943-016-0195-x . 26847152 10.1007/s10943-016-0195-x

[37] Jha AP, Stanley EA, Kiyonaga A, Wong L, Gelfand L. Examining the protective effects of mindfulness training on working memory capacity and affective experience. Emotion (Washington, DC). 2010;10(1):54–64. Epub 2010/02/10. doi: 10.1037/a0018438 . 20141302 10.1037/a0018438

[38] Lutz A, Slagter HA, Dunne JD, Davidson RJ. Attention regulation and monitoring in meditation. Trends in cognitive sciences. 2008;12(4):163–9. doi: 10.1016/j.tics.2008.01.005 18329323 10.1016/j.tics.2008.01.005 PMC2693206

[39] Wechsler D, Coalson DL, Raiford SE. WAIS-IV: Wechsler adult intelligence scale: Pearson San Antonio, TX; 2008.

[40] Frazier TW, Demaree HA, Youngstrom EA. Meta-analysis of intellectual and neuropsychological test performance in attention-deficit/hyperactivity disorder. Neuropsychology. 2004;18(3):543–55. Epub 2004/08/05. doi: 10.1037/0894-4105.18.3.543 . 15291732 10.1037/0894-4105.18.3.543

[41] Hale JB, Hoeppner J-AB, Fiorello CA. Analyzing digit span components for assessment of attention processes. Journal of Psychoeducational Assessment. 2002;20(2):128–43.

[42] Snyder HR, Miyake A, Hankin BL. Advancing understanding of executive function impairments and psychopathology: bridging the gap between clinical and cognitive approaches. Front Psychol. 2015;6:328 doi: 10.3389/fpsyg.2015.00328 25859234 10.3389/fpsyg.2015.00328 PMC4374537

[43] Brown KW, Ryan RM. The benefits of being present: mindfulness and its role in psychological well-being. Journal of personality and social psychology. 2003;84(4):822 12703651 10.1037/0022-3514.84.4.822

[44] Black DS, Sussman S, Johnson CA, Milam J. Psychometric Assessment of the Mindful Attention Awareness Scale (MAAS) Among Chinese Adolescents. Assessment. 2012;19(1):42–52. doi: 10.1177/1073191111415365 21816857 10.1177/1073191111415365 PMC3705937

[45] Jermann F, Billieux J, Laroi F, d'Argembeau A, Bondolfi G, Zermatten A, et al Mindful Attention Awareness Scale (MAAS): Psychometric properties of the French translation and exploration of its relations with emotion regulation strategies. Psychological assessment. 2009;21(4):506–14. Epub 2009/12/02. doi: 10.1037/a0017032 . 19947785 10.1037/a0017032

[46] Carlson LE, Brown KW. Validation of the Mindful Attention Awareness Scale in a cancer population. Journal of Psychosomatic Research. 2005;58(1):29–33. http://dx.doi.org/10.1016/j.jpsychores.2004.04.366. doi: 10.1016/j.jpsychores.2004.04.366 15771867 10.1016/j.jpsychores.2004.04.366

[47] MacKillop J, Anderson EJ. Further Psychometric Validation of the Mindful Attention Awareness Scale (MAAS). Journal of Psychopathology and Behavioral Assessment. 2007;29(4):289–93. doi: 10.1007/s10862-007-9045-1

[48] Estevis E, Basso MR, Combs D. Effects of practice on the Wechsler Adult Intelligence Scale-IV across 3- and 6-month intervals. The Clinical neuropsychologist. 2012;26(2):239–54. Epub 2012/02/23. doi: 10.1080/13854046.2012.659219 . 22353021 10.1080/13854046.2012.659219

[49] Waters AJ, Reitzel LR, Cinciripini P, Li Y, Marcus MT, Vidrine JI, et al Associations Between Mindfulness and Implicit Cognition and Self-Reported Affect. Substance abuse. 2009;30(4):328–37. doi: 10.1080/08897070903252080 19904668 10.1080/08897070903252080 PMC5024533

[50] Bishop SR, Lau M, Shapiro S, Carlson L, Anderson ND, Carmody J, et al Mindfulness: A Proposed Operational Definition. Clinical Psychology: Science and Practice. 2004;11(3):230–41. doi: 10.1093/clipsy.bph077

[51] Bhatia T, Mazumdar S, Wood J, He F, Gur RE, Gur RC, et al A randomised controlled trial of adjunctive yoga and adjunctive physical exercise training for cognitive dysfunction in schizophrenia. Acta neuropsychiatrica. 2016:1–13. Epub 2016/08/16. doi: 10.1017/neu.2016.42 . 27514629 10.1017/neu.2016.42 PMC5303681

[52] Gothe NP, Keswani RK, McAuley E. Yoga practice improves executive function by attenuating stress levels. Biological psychology. 2016;121(Pt A):109–16. Epub 2016/10/31. doi: 10.1016/j.biopsycho.2016.10.010 . 27794449 10.1016/j.biopsycho.2016.10.010

[53] Gothe NP, Kramer AF, McAuley E. The effects of an 8-week Hatha yoga intervention on executive function in older adults. The journals of gerontology Series A, Biological sciences and medical sciences. 2014;69(9):1109–16. Epub 2014/07/16. doi: 10.1093/gerona/glu095 ; PubMed Central PMCID: PMCPMC4202261. 25024234 10.1093/gerona/glu095 PMC4202261

[54] Hariprasad VR, Koparde V, Sivakumar PT, Varambally S, Thirthalli J, Varghese M, et al Randomized clinical trial of yoga-based intervention in residents from elderly homes: Effects on cognitive function. Indian J Psychiatry. 2013;55(Suppl 3):S357–63. Epub 2013/09/21. doi: 10.4103/0019-5545.116308 ; PubMed Central PMCID: PMCPMC3768212. 24049199 10.4103/0019-5545.116308 PMC3768212

[55] Brown RP, Gerbarg PL. Sudarshan Kriya Yogic breathing in the treatment of stress, anxiety, and depression. Part II—clinical applications and guidelines. Journal of alternative and complementary medicine (New York, NY). 2005;11(4):711–7. Epub 2005/09/01. doi: 10.1089/acm.2005.11.711 . 16131297 10.1089/acm.2005.11.711

[56] Chandra S, Sharma G, Sharma M, Jha D, Mittal AP. Workload regulation by Sudarshan Kriya: an EEG and ECG perspective. Brain informatics. 2016 Epub 2016/10/18. doi: 10.1007/s40708-016-0055-1 . 27747823 10.1007/s40708-016-0055-1 PMC5319952

[57] Leh SE, Petrides M, Strafella AP. The Neural Circuitry of Executive Functions in Healthy Subjects and Parkinson's Disease. Neuropsychopharmacology. 2010;35(1):70–85. doi: 10.1038/npp.2009.88 19657332 10.1038/npp.2009.88 PMC3055448

[58] Shepherd GMG. Corticostriatal connectivity and its role in disease. Nature reviews Neuroscience. 2013;14(4):278–91. doi: 10.1038/nrn3469 23511908 10.1038/nrn3469 PMC4096337

